# Sickness absence due to common mental disorders among precarious and non-precarious workers

**DOI:** 10.1093/eurpub/ckac130.127

**Published:** 2022-10-25

**Authors:** J Hernando-Rodriguez, N Matilla-Santander, M Almroth, B Kreshpaj, V Gunn, C Muntaner, T Bodin

**Affiliations:** 1 Unit of Occupational Medicine, Karolinska Institutet, Stockholm, Sweden; 2 Center for Social Data Science, University of Copenhagen, Copenhagen, Denmark; 3 MAP Centre for Urban Health Solutions, Li Ka Shing Knowledge Institute, Toronto, Canada; 4 Lawrence S. Bloomberg Faculty of Nursing, St. George Campus, Toronto, Canada; 5 Dalla Lana School of Public Health, University of Toronto, Toronto, Canada; 6 Department of Mental Health, The Johns Hopkins University Bloomberg School of Public Health, Baltimore, USA; 7 Centre for Occupational and Environmental Medicine, Stockholm Region, Stockholm, Sweden

## Abstract

**Background:**

Mental health disorders have become one of the leading diagnoses causing sickness absence. Previous studies have examined the impact of single employment characteristics or working conditions on sickness absence. However, few studies have investigated the effect of a multidimensional construct of precarious employment on sickness absence. Therefore, this study aims to describe sickness absence due to common mental disorders (CMD) as a proxy for access to social security benefits among precarious and non-precarious workers with mental health problems.

**Methods:**

Cohort register-based study of the total Swedish population aged 27 to 61 years residing in Sweden in 2016 and having mental health problems defined as being prescribed Selective Serotonin Reuptake Inhibitors (SSRI) in 2017 (N = 19,691). Individuals were classified as precariously employed or not based on a precarious employment score measured multidimensionally in 2016 (i.e., employment insecurity, income inadequacy, and lack of social protection). The outcome was the incidence of the first sickness absence episode due to CMD co-occurring with SSRI treatment in 2017. Logistic regression models will be performed.

**Results:**

The following results are preliminary. Precariously employed treated with SSRI were 8,68% in 2017. The distribution of a first sickness absence episode due to common mental disorders was similar in precarious and non-precarious workers (12.35% and 12.42%, respectively). Individuals directly employed (12.20%), with multiple jobs holding (14.62%), and low-medium income levels (14%) had higher sickness absence incidence due to common mental disorders. There were slight differences by gender.

**Conclusions:**

In these preliminary results, no differences were found between precarious and non-precarious workers with mental health problems in the distribution of sickness absence due to CMD. Further analysis will be conducted to investigate whether precarious employment is associated with sickness absences.

**Key messages:**

- Sickness absence due to common mental disorders is similarly distributed among precarious and non-precarious workers.
- Individuals with multiple jobs, low-income levels, and directly employed show higher sickness absence incidence due to common mental disorders.

